# Analysis of potential markers for detection of submicroscopic lymph node metastases in breast cancer

**DOI:** 10.1038/sj.bjc.6690636

**Published:** 1999-08

**Authors:** A E H Merrie, K Yun, J Gunn, L V Phillips, J L McCall

**Affiliations:** Departments of Surgery and Pathology, Dunedin School of Medicine, University of Otago, PO Box 913, Dunedin, New Zealand; Department of Surgery, University of Auckland, PO Box 92019, Auckland, New Zealand

**Keywords:** breast cancer, lymph node metastases, RT-PCR, molecular markers

## Abstract

We have developed sensitive assays for cytokeratin (K) 8, 16, 19, stromelysin 3 (ST3), MUC1 and maspin mRNAs using reverse transcription polymerase chain reaction (RT-PCR) and used these to assess lymph node status in patients undergoing surgery for breast cancer. In addition the RT-PCR assays were tested against lymph nodes from non-cancer patients to determine their specificity. Despite high sensitivity RT-PCR assays for K8, K16, K19, ST3 and maspin were not found to be useful as markers of submicroscopic disease as transcripts of these genes were detected in the great majority of control lymph nodes tested. Expression of MUC1 was also not found to be useful as it was both insensitive and non-specific. The importance of assessing potential markers against an adequately sized control population is demonstrated, as failure to do so can lead to erroneous conclusions. © 1999 Cancer Research Campaign


*British Journal of Cancer* (1999) **80**(12), 2019–2024
© 1999 Cancer Research Campaign
Article no. bjoc.1999.0636

### Analysis of potential markers for detection of submicroscopic lymph node metastases in breast cancer

**AEH Merrie[1], K Yun[2], J Gunn[1], LV Phillips[1] and JL McCall[3]**

Departments of [1]Surgery and [2]Pathology, Dunedin School of Medicine, University of Otago, PO Box 913, Dunedin, New Zealand; [3]Department of Surgery, University of Auckland, PO Box 92019, Auckland, New Zealand

**Summary** We have developed sensitive assays for cytokeratin (K) 8, 16, 19, stromelysin 3 (ST3), MUC1 and maspin mRNAs using reverse transcription polymerase chain reaction (RT-PCR) and used these to assess lymph node status in patients undergoing surgery for breast cancer. In addition the RT-PCR assays were tested against lymph nodes from non-cancer patients to determine their specificity. Despite high sensitivity RT-PCR assays for K8, K16, K19, ST3 and maspin were not found to be useful as markers of submicroscopic disease as transcripts of these genes were detected in the great majority of control lymph nodes tested. Expression of MUC1 was also not found to be useful as it was both insensitive and non-specific. The importance of assessing potential markers against an adequately sized control population is demonstrated, as failure to do so can lead to erroneous conclusions.

**Keywords**: breast cancer; lymph node metastases; RT-PCR; molecular markers

Following potentially curative surgery for breast cancer the presence of histologically evident tumour cells in the axillary lymph nodes is used to select high-risk patients for adjuvant therapy; however, 30% of histologically node-negative patients also develop metastatic disease. More accurate staging, in particular detection of occult metastatic disease, may enable effective treatment strategies to be extended to more high-risk patients.

Polymerase chain reaction (PCR) is up to 100 times more sensitive than conventional techniques in detecting circulating tumour cells and submicroscopic metastases (Ghossein and Rosai, 1996). However, the extreme sensitivity of PCR also confers an inherent disadvantage to produce false positive results. Furthermore, the central question of whether PCR-detected metastases reliably predicts relapse remains unanswered for many tumour types. Whilst lacking specific markers expressed by breast cancer cells a number of research groups have used cytokeratins 18 and 19, epithelial mucin (MUC1), carcinoembryonic antigen (CEA), CD 44 and maspin as transcript markers for the detection of submicroscopic metastases in lymph nodes, bone marrow or peripheral whole blood by reverse transcription PCR (RT-PCR) (Matsumura and Tarin, 1992; Datta et al, 1994; Gerhard et al, 1994; Noguchi et al, 1994, 1996*a*, 1996*b*; Schoenfeld et al, 1994, 1996, 1997; Brown et al, 1995; Mori et al, 1995; Gunn et al, 1996; Luppi et al, 1996; Yun et al, 1997; Eltahir et al, 1998; Lockett et al, 1998). However, there appears to be conflicting data regarding the specificity of some of these cell type specific markers in particular MUC-1 (Noguchi et al, 1994; Hoon et al, 1995), CD44 (Matsumura and Tarin, 1992; Eltahir et al, 1998) and K19 (Traweek et al, 1993; Schoenfeld et al, 1994, 1996; Burchill et al, 1995; Krismann et al, 1995; Gunn et al, 1996; Dingemans et al, 1997; Yun et al, 1997; Eltahir et al, 1998). Maspin expression has been previously reported as being a specific marker for breast cancer (Luppi et al, 1996); however, there have been no larger confirmatory studies to assess the accuracy and reproducibility of these findings. Although discrepancies in specificity may be attributed to RT-PCR methods employed including primer design, single-step PCR, two-stage PCR or signal detection by Southern blotting, clearly there is need to establish which markers may have potential in the diagnosis of minimal residual disease in breast cancer.

In an attempt to define a suitable cell type-specific marker for RT-PCR detection of submicroscopic lymph node metastases in breast cancer, we have examined a panel of candidate genes, with particular emphasis on sensitivity and specificity of gene expression in tissue from patients with and without breast cancer.

*Received 1 December 1998*
*Revised 2 February 1999*
*Accepted 16 February 1999*

*Correspondence to:* AEH Merrie

### MATERIALS AND METHODS

### Patients and tissue collection

Ethics committee approval to undertake this study was obtained. All patients undergoing either mastectomy or wide excision and axillary dissection were eligible for inclusion into the study. Fully informed written consent for collection of tissues was obtained. Mastectomy or wide excision specimens with axillary nodes were collected fresh from the operating theatre and lymph nodes were dissected prior to examination of the tumour on a clean UV irradiated chopping board with a sterile surgical blade to prevent epithelial cell contamination. Lymph nodes were bisected, with half submitted for routine histology and half taken for examination by RT-PCR. After lymph node dissection specimens of tumour and normal breast were taken from each specimen. Tumour grade

(Bloom and Richardson, 1957), size and the presence of ductal carcinoma in situ (DCIS) were recorded. Oestrogen and progesterone receptor status was assessed by immunohistochemistry using the 1D5 monoclonal and polyclonal antibody respectively (Dako). As a control group, lymph nodes were collected from patients undergoing surgery for histologically confirmed benign colorectal disease. Routine steps were taken in control specimens to avoid any luminal contamination prior to lymph node dissection.

### Markers

In common with other research groups we have developed sensitive assays for cytokeratins (K) 8 and 19 and MUC1 mRNAs using RT-PCR. In addition we have also investigated expression of stromelysin 3 (ST3), cytokeratin 16 (K16) and maspin. ST3 is a matrix metalloproteinase implicated in mammary carcinoma progression. In node-positive patients with infiltrating ductal carcinoma, multivariate analysis has revealed that ST3 level is a strong, independent prognostic parameter for disease-free survival (Ahmad et al, 1998). K16 has been reported as having expression limited to skin and breast tissue (Adams et al, 1995), making it a good potential marker of submicroscopic spread of breast cancer.

To control for the presence of epithelial cell contamination in the control population we used an assay for cytokeratin 20 (K20) which is a sensitive and specific gastrointestinal epithelial cell-specific marker (Gunn et al, 1996; Yun et al, 1997).

### Cell lines

Both MCF-7 and T47-D breast cancer cell lines were used to develop the assays. These cell lines are known to express MUC1 (Abe and Kufe, 1993) and K19 (Moll et al, 1982). Additionally MCF-7 has been reported to express maspin (Luppi et al, 1996). Both cell lines were grown and maintained in RPMI-1640 supplemented with 10% fetal calf serum at 37°C in 5% carbon dioxide.

### RNA extraction

Bisected lymph nodes and tissue samples from the resected specimen were collected into 1.5-ml microcentrifuge tubes containing 500 μl of 4 M guanidinium solution and manually homogenized using sterile DNA-free techniques. Total RNA from tissue samples and cell lines was extracted using a modification of the acid–guanidine isothiocyante–phenol–chloroform method (Chomczynski and Sacchi, 1987). RNA samples were measured by spectrophotometry at 260 nm and stored at –80°C until required. Rigorous steps were taken to avoid epithelial cell contamination, by physical separation of the component stages of specimen dissection, RNA extraction, cDNA synthesis, PCR and PCR product electrophoresis. All specimens were accompanied by a reagent only negative control.

### Reverse transcription

RNA samples were treated with RNase-free DNase 1 (Gibco BRL) prior to cDNA synthesis. cDNA was synthesized from a maximum of 2.5 μg of DNase-treated total RNA, with 200 units of M-MLuV Reverse Transcriptase (GibcoBRL) primed with random hexamers (Boehringer Mannheim), using the manufacturer's method in a total volume of 10 μl. Reverse transcriptase minus controls were prepared for each DNase-treated RNA sample. One-twentieth of synthesized cDNA was used for PCR.

### Oligonucleotide primers

Custom PCR primers (GibcoBRL) were designed as follows:

| | |
|---|---|
| K8 F | GCG GCA GCT GCG TGA GTA |
| K8 R | GCT GAG GCC GGG GCT TGT GAG |
| K16 outer F | TCA ATG ACC GCC TGG CCT CTT A |
| K16 outer R | CAG GGC CAG TTC GTG CTC ATA |
| K16 inner F | CAA CGC CGA CCT GGA AGT G |
| K16 inner R | CAA TGG TGG CCG CAA TGA T |
| K19 F | CCA AGA TCC TGA GTG ACA TGC GAA G |
| K19 R | TGC AGC TCA ATC TCA AGA CCC TGA A |
| Maspin F | CAA GTG GGT GCT AAA GGT GAC |
| Maspin R | CAA AGT GGC CAT CTG TGA G |
| MUC1 F | CGT CGT GGA CAT TGA TCC TAC C |
| MUCI R | GGT ACC TCC TCT CAC CTC CTC CAA |
| ST3 F | GGC GTG CCC GAC CCA TCT |
| ST3 R | CGG CCC TCG TGC ACC TCA GTA A |
| K20 F | AGA CCA AGG CCC GTT ACA G |
| K20 R | ACG ACC TTG CCA TCC ACT ACT TC |

K19 primers were designed to span exons 4 and 5, hence spanning the shortest intron of the gene sequence, as well as incorporating mismatches between the pseudogene and cDNA sequences in the 3′ pentamers (Bader et al, 1988; Gunn et al, 1996). K16 primers were designed to span exons 1 and 3 at the 5′ end thus avoiding the known sequence of K16 pseudogene, with nested primers designed to span exons 1 and 2. β-actin PCR was performed on each specimen as an endogenous external control for RNA extraction and cDNA synthesis.

### Polymerase chain reaction

PCR was carried out in a Hybaid Touchdown PCR machine (Hybaid, Middlesex, UK) with an initial denaturation at 94°C and final extension at 72°C common to all PCR reactions. Individual assays were as follows:

| | |
|---|---|
| K8: | 35 cycles of 94°C 30 s, 61°C 30 s, 72°C 30 s |
| K16 outer: | 40 cycles of 94°C 30 s, 59°C 30 s, 72°C 30 s |
| K16 nested: | 35 cycles of 94°C 30 s, 59°C 30 s, 72°C 30 s |
| K19: | 35 cycles of 94°C 30 s, 62°C 30 s, 72°C 30 s |
| Maspin: | 35 cycles of 94°C 30 s, 50°C 30 s, 72°C 30 s |
| MUC1: | 40 cycles of 94°C 30 s, 59°C 30 s, 72°C 30 s |
| ST 3: | 35 cycles of 94°C 30 s, 56°C 30 s, 72°C 30 s |
| K20: | 35 cycles of 94°C 30 s, 62.5°C 30 s, 72°C 30 s |

The PCR mix consisted of 150 μM of each dNTP, 1 μM of each primer, 1 × PCR buffer (Qiagen) and 0.5 units of *Taq* DNA polymerase (Qiagen) in a total volume of 10 μl. All PCR assays included a DNA-positive control and a no-template negative control. Five microlitre aliquots of the resulting PCR products were examined on 0.5 μg $ml^{-1}$ ethidium bromide stained, 2% agarose/TAE gels for presence or absence of PCR products.

### Analysis

Sensitivity of the assays was calculated on the group of patients with histologically proven breast cancer by comparison of patients with histologically evident lymph node metastases and patients

*© 1999 Cancer Research Campaign*

**Table 1** Patients with histologically confirmed breast cancer

| Marker | Number of patients | Histologically positive (%) | Marker positive (%) | Sensitivity of marker % (95% CI) |
|---|---|---|---|---|
| K 8 | 36 | 13 (36%) | 35 (97%) | 100 (75.3–100) |
| K 16 | 53 | 22 (42%) | 38 (72%) | 100 (84.6–100) |
| K 19 | 36 | 13 (36%) | 35 (97%) | 100 (75.3–100) |
| Maspin | 9 | 9 (100%) | 9 (100%) | 100 (66.4–100) |
| MUC 1 | 18 | 10 (56%) | 11 (61%) | 60 (26.2–87.8) |
| ST 3 | 39 | 15 (38%) | 37 (95%) | 100 (78.2–100) |

**Table 2** Lymph nodes from patients with breast cancer

| Marker | Number of lymph nodes | Histologically positive nodes (%) | Marker positive nodes (%) |
|---|---|---|---|
| K 8 | 313 | 56 (18%) | 255 (81%) |
| K 16 | 476 | 90 (19%) | 108 (23%) |
| K 19 | 313 | 56 (18%) | 221 (71%) |
| Maspin | 90 | 66 (73%) | 50 (55%) |
| MUC 1 | 159 | 41 (26%) | 28 (18%) |
| ST 3 | 342 | 69 (20%) | 239 (70%) |

**Table 3** Non-cancer control patients

| Marker | Number of patients | Marker positive | Specificity of marker % (95% CI) |
|---|---|---|---|
| K 8 | 8 | 8 | 0 (0–36.9) |
| K 16 | 22 | 10 | 54.5 (32.2–75.6) |
| K 19 | 11 | 9 | 18.2 (2.3–51.8) |
| K 20 | 35 | 0 | 100 (90.0–100) |
| Maspin | 13 | 11 | 15.4 (1.9–45.5) |
| MUC 1 | 10 | 4 | 60 (26.2–87.8) |
| ST 3 | 9 | 9 | 0 (0–33.6) |

**Table 4** Lymph nodes from non-cancer patients

| Marker | Number of lymph nodes | Marker positive nodes (%) |
|---|---|---|
| K 8 | 54 | 52 (96%) |
| K 16 | 146 | 26 (18%) |
| K 19 | 61 | 41 (67%) |
| K 20 | 249 | 0 (0%) |
| Maspin | 113 | 54 (48%) |
| MUC 1 | 90 | 44 (49%) |
| ST 3 | 64 | 41 (64%) |

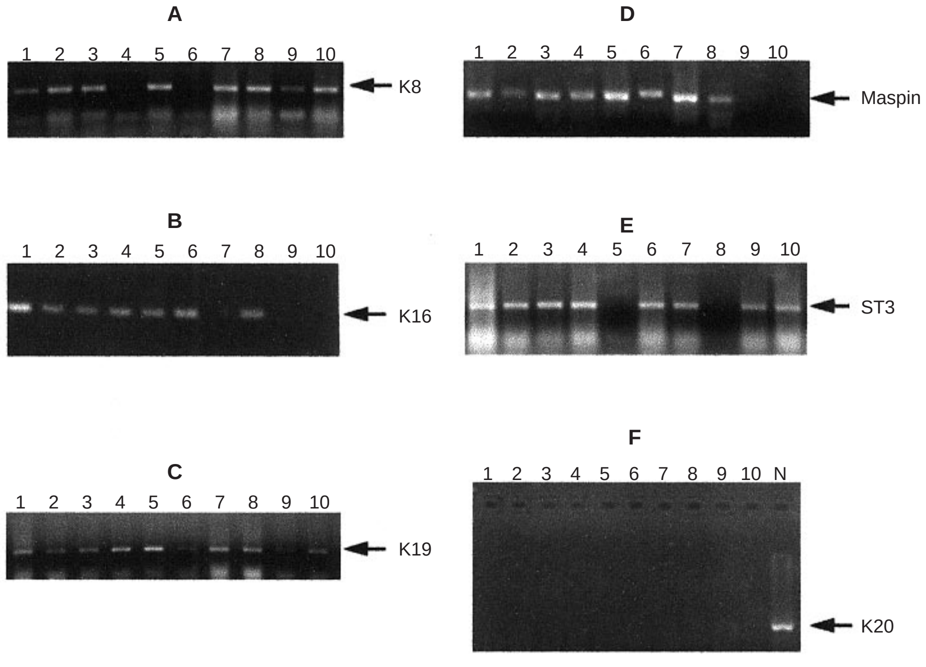

**Figure 1** Ethidium bromide stained agarose gels of RT-PCR products of potential markers for detection of submicroscopic spread of breast cancer. Lanes 1–10 represent ten lymph nodes from a patient without cancer (**A**) K8, (**B**) K16, (**C**) K19, (**D**) Maspin, (**E**) ST3. (**F**) K20, demonstrating lack of epithelial cell contamination in the control lymph nodes with PCR product present only in the normal colonic epithelia (Lane N)

with marker-positive disease. Specificity was determined against patients with benign colorectal conditions confirmed on histology. As all assays were expected to be more sensitive than histology, specificity could only truly be determined by comparison against a population with no histological evidence of cancer. For both sensitivity and specificity 95% confidence intervals (95% CI) were used to control for sample size (Merrie et al, 1998) as calculated using CIA software (Gardner and Altman, 1989).

### RESULTS

RT-PCR assays for K8, K16, K19, maspin and ST 3 were found to be 100% sensitive compared to histology (Table 1), but with considerable variation in the proportion of positive lymph nodes from patients with breast cancer (Table 2). However, these markers were not specific (Table 3), with transcripts of these genes detected in many of the control lymph node samples tested

*© 1999 Cancer Research Campaign*

**Table 5** Control patients and results from reported breast markers

| Reference | Marker | Tissue | Number of patients | Marker positive | Specificity of marker % (95% CI) |
|---|---|---|---|---|---|
| Matsumura and Tarin, 1992 | CD44 | PBL | 4 | 0 | 100 (39.8–100) |
| Eltahir et al, 1998 | CD44 | PBL | 10 | 4 | 60 (26.2–87.8) |
| Gerhard et al, 1994 | CEA | BM/PBL | 56 | 0 | 100 (93.6–100) |
| Mori et al, 1995 | CEA | LN | 5 | 0 | 100 (47.8–100) |
| Brown et al, 1995 | DF 3 | PBL | 4 | 0 | 100 (39.8–100) |
| Brown et al, 1995 | K 18 | PBL | 4 | 4 | 0 (0–60.2) |
| Schoenfeld et al, 1994 | K 19 | LN | 11 | 0 | 100 (71.5–100) |
| Datta et al, 1994 | K 19 | PBL | 10 | 0 | 94.9 (82.7–99.4) |
| | | BM | 29 | 2 | |
| Schoenfeld, 1996 | K 19 | LN | 20 | 0 | 100 (83.2–100) |
| Luppi et al, 1996 | K 19 | PBL | 17 | 5 | 70.6 (44–89.7) |
| Noguchi et al, 1996*a* | K 19 | LN | (10)* | (0)* | – |
| Schoenfeld et al, 1997 | K 19 | PBL/BM | 25 | 0 | 100 (86.3–100) |
| Eltahir et al, 1998 | K 19 | PBL | 5 | 0 | 100 (47.8–100) |
| Lockett et al, 1998 | K 19 | LN | 9 | 0 | 100 (66.4–100) |
| | c-myc | | | | |
| | PIP | | | | |
| Luppi et al, 1996 | Maspin | PBL | 17 | 0 | 100 (83.9–100) |
| | | BM | 4 | | |
| Noguchi et al, 1994 | MUC 1 | LN | (10)* | (0)* | – |
| Hoon et al, 1995 | MUC 1 | PBL | 8 | 7 | 12.5 (3.1–52.7) |
| | | LN | (8)* | (4)* | |
| Eltahir et al, 1998 | MUC 1 | PBL | 23 | 21 | 8.7 (1.1–28.0) |

Specificity of previously reported markers for the detection of submicroscopic spread of breast cancer. BM, bone marrow; LN, lymph nodes; PBL, peripheral blood leucocytes. *number of lymph nodes only, patient numbers not reported.

(Table 4, Figure 1). Expression of MUC1 was found to be both poorly sensitive and non-specific. K20 expression was not evident in any of the control lymph nodes assessed (Tables 3 and 4).

Initial assessment of K16 using a limited number of controls showed only two out of 52 lymph nodes from eight patients to be K16 RT-PCR-positive, however, extension of the control assessment to 146 nodes from 22 patients showed that 26 lymph nodes in ten patients without epithelial malignancy were K16 RT-PCR-positive (Table 3).

### DISCUSSION

There have been several studies attempting to define markers for the detection of submicroscopic disease in breast cancer, with apparently convincing results for CEA, K19 and maspin (Table 5). However, data from studies assessing the detection of disease in colon and lung cancer have raised questions with regard to the tissue specificity of these markers (Adams et al, 1995; Burchill et al, 1995; Hoon et al, 1995; Krismann et al, 1995; Denis et al, 1997; Dingemans et al, 1997; Eltahir et al, 1998)

CEA gene expression must be regarded with caution as a tumour specific marker, as it has been detected by nested RT-PCR in normal tissues. Jonas et al (1996) found 23% of controls without cancer had evidence of CEA expression in peripheral blood, which the authors propose may be due to venepuncture-induced skin contamination. More recently, Liefers et al (1998) used the nested CEA RT-PCR assay developed by Gerhard et al (1994) for the detection of disease in lymph nodes. However, only seven lymph nodes from two patients were used as controls and CEA expression was detected in these at high cycle numbers. These results raise doubt with regards to the 100% tissue specificity reported by Gerhard et al (1994) (Table 5).

The results from this study confirm a lack of tissue specificity for both MUC-1 and K19 gene expression. Two previous reports have suggested MUC-1 to be tissue specific (Noguchi et al, 1994, 1996*a*); however, there is no reported data on the number of control patients, and a total of only ten lymph nodes assessed. Our results concur with that of other groups in showing that MUC-1 is expressed in cells of lymphohaemopoetic origin (Hoon et al, 1995; Eltahir et al, 1998). Several studies in breast cancer have reported K19 to be a tissue-specific marker (Datta et al, 1994; Schoenfeld et al, 1994, 1996, 1997; Noguchi et al, 1996*a*; Eltahir et al, 1998; Lockett et al, 1998). However, there is now a substantial body of evidence to show that K19 can also be detected in peripheral blood and lymphatic tissue rendering it unsuitable as a specific marker of submicroscopic disease (Adams et al, 1995; Burchill et al, 1995; Krismann et al, 1995; Denis et al, 1997; Dingemans et al, 1997).

In contrast to Luppi et al (1996) we did not find that maspin proved to be a specific marker of occult tumour spread in breast cancer. Using the primers and conditions reported by Luppi et al, the maspin gene product could not be amplified in the T47-D and MCF-7 cell lines or in genomic DNA. Analysis of these primers revealed a 5 bp (base pair) self-dimer at the 3´ end of the forward primer, a double 3 bp self-dimer in the reverse primer and a 4 bp pair dimer between the two primers, resulting in marked primer dimer formation and poorly specific priming of the maspin gene. Custom PCR primers (Gibco BRL) were subsequently redesigned using PrimerSelect (DNASTAR) with considerably less primer dimer formation and straightforward amplification of the maspin gene product. Using the redesigned primers maspin gene expression was found to vary markedly between tumours, and was evident in many control nodes. In addition to this the MCF-7 cell line did not display evidence of maspin expression (Figure 2).

 © 1999 Cancer Research Campaign

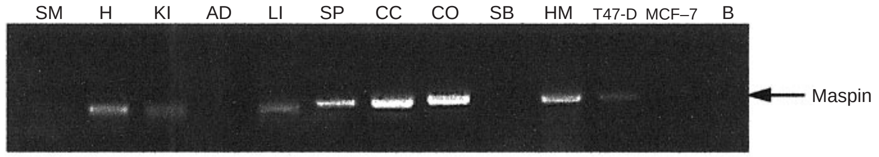

**Figure 2** Tissue specificity of maspin RT-PCR product, demonstrating a lack of tissue specificity and absence of expression in the MCF-7 cell line. SM, skeletal muscle; H, heart; KI, kidney; AD, adrenal; LI, normal liver; SP, spleen; CC, colon adenocarcinoma; CO, normal colon; SB, small bowel; HM, hepatic metastasis; T47-D and MCF-7, breast cancer cell lines; B, PCR control

Although K16 expression appeared less ubiquitous compared to other genetic markers, it also lacked specificity, as did ST3 and K8. With regards to K16, assessment of only ten lymph nodes as in previous studies (Noguchi et al, 1994, 1996*a*), would have resulted in a false assertion of 100% specificity. However, when the number of control nodes was increased to 154 from 23 patients, a more accurate determination of specificity was possible.

Although many previous studies have reported 100% specificity, analysis of 95% confidence intervals (Table 5) shows that the majority cannot make this claim with any degree of certainty. Use of a large control group increases the reliability of the determination of specificity, and examination of too few patients can result in false estimates.

We have adopted rigorous protocols to avoid and monitor for epithelial cell contamination. This is achieved by strategically separating component parts of the RT-PCR assay, the use of sterile DNA-free techniques, disposable consumables, reagent only negative controls, reverse transcriptase minus controls and PCR reagent only controls. The adherence to such methodology is essential for success of any RT-PCR assay. The absence of K20 in the control lymph nodes rules out the possibility of epithelial cell contamination from the gastrointestinal tract, confirming the validity of the positive results of the potential markers.

To date there have been no markers of submicroscopic spread of breast cancer identified that are both sensitive and specific. Many markers such as K19 and maspin have been initially reported as sensitive and specific, but little emphasis has been placed on determining assay specificity to a reliable level.

Markers of submicroscopic spread in breast cancer could have potential therapeutic impact, especially when combined with sentinel node assessment of axillary disease. As yet no such marker exists and future assays must be assessed by the use of control populations of sufficient size to reliably determine specificity as well as sensitivity.

### ACKNOWLEDGEMENTS

We would like to acknowledge the Health Research Council of New Zealand and the Anderson Telford Trust for their financial support. We are also indebted to Noel Gordon-Glassford, Penny Wright, Kerrie Knapp, Shona McDowell and Michael Watson for their skill and patience in obtaining the tissue samples.

### REFERENCES

Abe M and Kufe D (1993) Characterization of cis-acting elements regulating transcription of the human DF3 breast carcinoma-associated antigen (MUC1) gene. *Proc Natl Acad Sci USA* **90**: 282–286

Adams MD, Kerlavage AR, Fleischmann RD, Fuldner RA, Bult CJ, Lee NH, Kirkness EF, Weinstock KG, Gocayne JD, White O, Sutton G, Blake JA, Brandon RC, Chin M, Clayton RA, Cline RT, Cotton MD, Earle-Hugh J, Fine LD, Fitzgerald LM, Fitzhugh WM, Fritchman JL, Geoghagen NSM, Glodek A, Gnehm CL, Hanna MC, Medblom E, Hinkle PS, Kelley JM, Klimek KM, Kelley JC, Lin L, Marmaros SM, Merrick JM, Moren-Palanques RF, McDonald LA, Ngugen DT, Pellegrino SM, Phillips CA, Ryder SE, Scott JL, Saudek DM, Shirley R, Small KV, Spriggs TA, Utterback TR, Weidman JF, Li Y, Barthlow R, Bednarik DP, Cao L, Cepeda MA, Coleman TA, Collins E, Dimke D, Feng P, Ferrie A, Fischer C, Hastings GA, He W, Hu J, Huddleston KA, Greene JM, Gruber J, Hudson P, Kim A, Kozak DL, Kunsch C, Ji H, Li H, Meissner PS, Olsen H, Raymond L, Wei Y, Wing J, Xu C, Yu G, Ruben SM, Dillon PJ, Fannon MR, Rosen CA, Haseltine LA, Fields C, Fraser CM and Ventner JC (1995) Initial assessment of human gene diversity and expression patterns based upon 83 million nucleotides of cDNA sequence. *Nature* **377**: 3–174

Ahmad A, Hanby A, Dublin E, Poulsom R, Smith P, Barnes D, Rubens R, Anglard P and Hart I (1998) Stromelysin 3: an independent prognostic factor for relapse-free survival in node-positive breast cancer and demonstration of novel breast carcinoma cell expression. *Am J Pathol* **152**: 721–728

Bader BL, Jahn L and Franke WW (1988) Low level expression of cytokeratins 8, 18 and 19 in vascular smooth muscle cells of human umbilical cord and in cultured cells derived therefrom, with an analysis of the chromosomal locus containing the cytokeratin 19 gene. *Eur J Cell Biol* **47**: 300–319

Bloom HJG and Richardson WW (1957) Histological grading and prognosis in breast cancer. *Br J Cancer* **11**: 359–364

Brown DC, Purushotham AD, Birnie GD and George WD (1995) Detection of intraoperative tumor cell dissemination in patients with breast cancer by use of reverse transcription and polymerase chain reaction. *Surgery* **117**: 96–101

Burchill SA, Bradbury MF, Pittman K, Southgate J, Smith B and Selby P (1995) Detection of epithelial cancer cells in peripheral blood by reverse transcriptase-polymerase chain reaction. *Br J Cancer* **71**: 278–281

Chomczynski P and Sacchi N (1987) Single step method of RNA isolation by acid guanidinium thiocyanatephenol-chloroform extraction. *Anal Biochem* **162**: 156–159

Datta YH, Adams PT, Drobyski WR, Ethier SP, Terry VH and Roth MS (1994) Sensitive detection of occult breast cancer by the reverse-transcriptase polymerase chain reaction. *J Clin Oncol* **12**: 475–482

Denis MG, Lipart C, Leborgne J, Lehur PA, Galmiche JP, Denis M, Ruud E, Truchaud A and Lustenberger P (1997) Detection Of Disseminated Tumor Cells In Peripheral Blood Of Colorectal Cancer Patients. *Int J Cancer* **74**: 540–544

Dingemans A-MC, Brakenhoff RH, Postmus PE and Giaccone G (1997) Detection of cytokeratin-19 transcripts by reverse transcriptase-polymerase chain reaction in lung cancer cell lines and blood of lung cancer patients. *Lab Invest* **77**: 213–220

Eltahir EM, Mallinson DS, Birnie GD, Hagan C, George WD and Purushotham AD (1998) Putative markers for the detection of breast carcinoma cells in blood. *Br J Cancer* **77**: 1203–1207

Gardner MJ and Altman DG (1989) *Statistics with Confidence – Confidence Intervals and Statistical Guidelines*. BMJ Publishing: London

Gerhard M, Juhl H, Kalthoff H, Schreiber HW, Wagener C and Neumaier M (1994) Specific detection of carcinoembryonic antigen-expressing tumor cells in bone marrow aspirates by polymerase chain reaction. *J Clin Oncol* **12**: 725–729

Ghossein RA and Rosai J (1996) Polymerase chain reaction in the detection of micrometastases and circulating tumor cells. *Cancer* **78**: 10–16

Gunn J, McCall JL, Yun K and Wright PA (1996) Detection of micrometastases in colorectal cancer patients by K19 and K20 reverse-transcription polymerase chain reaction. *Lab Invest* **75**: 611–616

Hoon DS, Doi F, Giuliano AE, Schmid P and Conrad AJ (1995) The detection of breast carcinoma micrometastases in axillary lymph nodes by means of reverse transcriptase-polymerase chain reaction. *Cancer* **76**: 533–535

Jonas S, Windeatt S, O-Boateng A, Fordy C and Allen-Mersh TG (1996) Identification of carcinoembryonic antigen-producing cells circulating in the blood by reverse transcriptase polymerase chain reaction. *Gut* **39**: 717–721

© 1999 Cancer Research Campaign 

Krismann M, Todt B, Schroder J, Gareis D, Muller KM, Seeber S and Schutte J (1995) Low specificity of cytokeratin 19 reverse transcriptase-polymerase chain reaction analyses for detection of hematogenous lung cancer dissemination. *J Clin Oncol* **13**: 2769–2775

Liefers J-G, Cleton-Jansen AM, van de Velde CJH, Hermans J, van Krieken JHJM, Cornelisse CJ and Tollenaar RAEM (1998) Micrometastases and survival in stage II colorectal cancer. *N Engl J Med* **339**: 223–228

Lockett MA, Baron PL, O'Brien PH, Elliott BM, Robison JG, Maitre N, Metcalf JS and Cole DJ (1998) Detection of occult breast cancer micrometastases in axillary lymph nodes using a multimarker reverse transcriptase-polymerase chain reaction panel. *J Am Col Surg* **187**: 9–16

Luppi M, Morselli M, Bandieri E, Federico M, Marasca R, Barozzi P, Ferrari MG, Savarino M, Frassoldati A and Torelli G (1996) Sensitive detection of circulating breast cancer cells by reverse-transcriptase polymerase chain reaction of maspin gene. *Annal Oncol* **7**: 619–624

Matsumura Y and Tarin D (1992) Significance of CD44 gene products for cancer diagnosis and disease evaluation. *Lancet* **340**: 1053–1058

Merrie AEH, Yun K and McCall JL (1998) Detection of carcinoembryonic antigen messenger RNA in lymph nodes from patients with colorectal cancer. *N Engl J Med* **339**: 1642–1644

Moll R, Franke WW, Schiller DL, Geiger B and Krepler R (1982) The catalog of human cytokeratins: patterns of expression in normal epithelia, tumors and cultured cells. *Cell* **31**: 11–24

Mori M, Mimori K, Inoue H, Barnard GF, Tsuji K, Nanbara S, Ueo H and Akiyoshi T (1995) Detection of cancer micrometastases in lymph nodes by reverse transcriptase-polymerase chain reaction. *Cancer Res* **55**: 3417–3420

Noguchi S, Aihara T, Nakamori S, Motomura K, Inaji H, Imaoka S and Koyama H (1994) The detection of breast carcinoma micrometastases in axillary lymph nodes by means of reverse transcriptase-polymerase chain reaction. *Cancer* **74**: 1595–1600

Noguchi S, Aihara T, Motomura K, Inaji H, Imaoka S and Koyama H (1996*a*) Detection of breast cancer micrometastases in axillary lymph nodes by means of reverse transcriptase-polymerase chain reaction. Comparison between MUC1 mRNA and keratin 19 mRNA amplification. *Am J Path* **148**: 649–656

Noguchi S, Aihara T, Motomura K, Inaji H, Imaoka S and Koyama H (1996*b*) Histologic characteristics of breast cancers with occult lymph node metastases detected by keratin 19 mRNA reverse transcriptase polymerase chain reaction. *Cancer* **78**: 1235–1240

Schoenfeld A, Luqmani Y, Smith D, O'Reilly S, Shousha S, Sinnett HD and Coombes RC (1994) Detection of breast cancer micrometastases in axillary lymph nodes by using polymerase chain reaction. *Cancer Res* **54**: 2986–2990

Schoenfeld A, Luqmani Y, Sinnett HD, Shousa S and Coombes RC (1996) Keratin 19 mRNA measurement to detect micrometastases in lymph nodes in breast cancer patients. *Br J Cancer* **74**: 1639–1642

Schoenfeld A, Kruger KH, Gomm J, Sinnett HD, Gazet JC, Sacks N, Bender HG, Luqmani Y and Coombes RC (1997) The detection of micrometastases in the peripheral blood and bone marrow of patients with breast cancer using immunohistochemistry and reverse transcriptase polymerase chain reaction for keratin 19. *Eur J Cancer* **33**: 854–861

Traweek ST, Liu J and Battifora H (1993) Keratin gene expression in non-epithelial tissues. Detection with polymerase chain reaction. *Am J Pathol* **142**: 1111–1118

Yun K, Gunn J, Merrie A, Phillips LV and McCall JL (1997) Keratin 19 is detectable by RT-PCR in lymph nodes of patients with breast cancer. *Br J Cancer* **76**: 1112–1113

 *© 1999 Cancer Research Campaign*
